# Hybrid Multi-Level Detection and Mitigation of Clone Attacks in Mobile Wireless Sensor Network (MWSN)

**DOI:** 10.3390/s20082283

**Published:** 2020-04-17

**Authors:** Haafizah Rameeza Shaukat, Fazirulhisyam Hashim, Muhammad Arslan Shaukat, Kamal Ali Alezabi

**Affiliations:** 1 Department of Computer and Communication Systems Engineering, Universiti Putra Malaysia, Selangor 43400, Malaysia; 2 School of Engineering and Information Technology, University of Technology Sydney, Sydney 2007, Australia; marslanshaukat@gmail.com; 3 School of Information Technology, FoBIS, UCSI University, Kula Lumpur 56000, Malaysia; kamal@ucsiuniversity.edu.my

**Keywords:** mobile wireless sensor network, hybrid, node replication attack, danger theory, danger zone, wireless sensor network

## Abstract

Wireless sensor networks (WSNs) are often deployed in hostile environments, where an adversary can physically capture some of the sensor nodes. The adversary collects all the nodes’ important credentials and subsequently replicate the nodes, which may expose the network to a number of other security attacks, and eventually compromise the entire network. This harmful attack where a single or more nodes illegitimately claims an identity as replicas is known as the node replication attack. The problem of node replication attack can be further aggravated due to the mobile nature in WSN. In this paper, we propose an extended version of multi-level replica detection technique built on Danger Theory (DT), which utilizes a hybrid approach (centralized and distributed) to shield the mobile wireless sensor networks (MWSNs) from clone attacks. The danger theory concept depends on a multi-level of detections; first stage (highlights the danger zone (DZ) by checking the abnormal behavior of mobile nodes), second stage (battery check and random number) and third stage (inform about replica to other networks). The DT method performance is highlighted through security parameters such as false negative, energy, detection time, communication overhead and delay in detection. The proposed approach also demonstrates that the hybrid DT method is capable and successful in detecting and mitigating any malicious activities initiated by the replica. Nowadays, crimes are vastly increasing and it is crucial to modify the systems accordingly. Indeed, it is understood that the communication needs to be secured by keen observation at each level of detection. The simulation results show that the proposed approach overcomes the weaknesses of the previous and existing centralized and distributed approaches and enhances the performance of MWSN in terms of communication and memory overhead.

## 1. Introduction

Currently, the major focus of research has been on mobile wireless sensor networks (MWSNs), as an advantage of mobile nodes that control the movement by itself. Further, it has been noticed that mobility reduces the problems regarding maximum coverage area and connectivity. However, in MWSNs, the most important task is the position estimation of mobile nodes. Recently, many applications for execution mobility perform an important role in a specific type of Wireless Sensor Network (WSN), which are known as MWSNs. Currently, MWSN has become the main focus of research as it has many issues regarding connectivity, coverage area and energy consumption. Recent research has shown that the features of mobility reduce many problems instead of making them complex. Nowadays, MWSNs are used in many applications like monitoring, tracking and sensing in different environments. Therefore, mobile nodes are important and necessary modules in a wide range of applications of sensor networks [1]. Typically, the wireless sensor nodes are unattended that contains mobile nodes with low cost features. Mobile nodes are comprised of multiple components, including mobility, memory, power, micro-controller, sensing capabilities and communication features. Nowadays, there are numerous types of security attacks identical to wormhole, clone attacks, DoS, cyber security, routing, Sybil, jamming, privacy factor, physical, black hole and others. A clone node attack is a perilous one in MWSN. An advisory can grab the mobile node in order to capture the information stored in it and later on can generate manifold clones of the captured mobile node. Thus, a clone attack eventually depends on how briskly a malicious activity can initiate in the network. An advisory conquers the cons of the unattended nature of compromised node and attains the whole data from its memory such as its key, identity, communicating information and so on. Additionally, an advisory can imitate the captured mobile node and deploy it again back to the network in the target destination. Afterwards, this compromise mobile node can be used by the advisory to monitor and control the multiple features. Therefore, it can be very harmful if the clone node is not detected on time.

In this paper, our major concern is to explore the concept of Danger Theory (DT) for clone node detection. In the primary work [2], we recruited the solution of clone detection which relies upon two methods, specifically, attack detection and security control with respect to the true positive, false negative and false positive rate. A motivation of the proposed enhanced version of the DT method supports the identification of a clone in a secure manner. The multi-steps detection approach is comprised of three stages of recognition; first step (based upon clustering method), second step (correlation of the battery level and random numbers) and third (secure network). For detecting the existence of malicious nodes, the proposed DT method implements the concept of clone detection with multiple stages. The extended version is proposed in this paper to secure the communication from duplicate nodes with addition to password protection. It is crucial to effectively secure the nodes’ credentials in terms of communication overhead, frequency, energy, detection rate, life time, delay and false negative. In this paper, the advanced experiments are originated to sort out the weaknesses of previous works [2]. With merging the idea of password protection approach, the security experimental results achieved are more accurate and precise. Furthermore, we have provided the comparison of the proposed method to other existing methods. We observed interesting differences between the DT approach and existing methods, whereby each level shows a unique and efficient analysis of clone detection. Therefore, in this paper, the significances of both methods’ voltage comparison and password protection are executed and evaluated.

The rest of the proposed paper is classified as follows. Section 2 briefly elaborates the existing methods for detecting clone mobile nodes. Section 3 highlights the secure DT based work in terms of network and threat model in MWSN. In addition, it also discusses about the multi-steps DT approach more briefly with different features. Section 4 focuses on the process of the clone detection, precisely the first step (clustering), second step (correlation of the battery level and random numbers) and third step (network security) which are demonstrated and described in detail. The performance evaluation and discussion of the outcomes are simulated in Section 5 and Section 6, respectively, and the proposed method is concluded in Section 7.

## 2. Existing Replica Detection Methods

In MWSN, mobile nodes are performing important functions and multi orientated tasks in various applications. Moreover, it is comprised of numerous factors and can take additional benefits as compared to static node [3]. Existing approaches for detection and mitigation clone threats are categorized in two categories, which are distributed and centralized.

### 2.1. Centralized Methods

This method depends on the speed of legitimate nodes which must be less than the network speed, and the clone will travel much quicker than the network maximum speed. Hence, the mobile node speed limit must be checked and compared. If a speed limit increases the network speed, at that moment there can be a chance that the mobile nodes of the same identity are existing [4,5]. In [6], a novel protocol for the recognition of clone threat elaborated. This theory is created on the concept of Polynomial-based pair-wise key pre-distribution and Bloom Filters, which explained that the clone node cannot deploy into real presence and pair wise keys of each mobile node in MWSN. Hence, replicas can be known by computing the predefined value, and is identified when it goes beyond the settled value.

### 2.2. Distributed Methods

The Efficient and Distributed Detection (EDD) and Storage Efficient and Distributed Detection (SEDD) [7] rely on factors such as the times interval, whereby when one node encounters another mobile node, it must be less than the mentioned interval. The EDD method relies on two stages, namely online and offline. The first step is simulated earlier to deployment and another step is initiated on per move of every node in MWSN. The SEED method tackles the overhead storage in MWSN and gives the solution of time interval of EDD. Hence, each node only screens the subset rather than the whole in precise time interval. Two scenarios have been elaborated by Patrol detection [8], namely, static and mobile nodes for clone attacks. It depends on the perception that there is a chance of clone mobile node if two or several nodes have same identity or key at various location. Moreover, speed limit check on mobile node, if increase the certain limit will be a chance of clone node in MWSN. Onward that mobile node will access the distance and correlate it with range and later save the information in white and black lists where black list identified as clone identity. Therefore, if Patroller fluctuates the position, it moves in different time interval i.e., *T*, *T* + Interval. The Single Hop Detection (SHD) [9] method proposed for clone states that nodes in MWSN at a specific time cannot exist in neighbor node list. The existing method depends upon the fingerprint verification and claim. Consequently, two mobile nodes interchange the information i.e., witness node and when two claims with the same identity frequency matches in the neighbor group, it indicates the existence of a clone. The token exchange depends on the deployed mobile nodes do not cooperate in clone approach has been proposed in [10]. Therefore, during mobile node frequency of meeting with other mobile node exchange, the tokens are stored in their memory. On the next meeting, each node will ask about the last exchanged token and wrong information will specify as the clone node. Moreover, they also explained that the idea relies on each node’s cooperation, and same node presence seen multiple times are also known as clone nodes. In [11], the authors proposed two solutions for clone detection, namely SDD (Simple Distributed Detection) and CDD (Cooperative Distributed Detection). It relies on the number of observations of node meeting, where it is necessary to re-meet the other node in a precise time period otherwise the node would be considered as a clone node. The CDD approach is initiated for more collaboration in clone detection. The UTLSE (Unary Time Location Storage and Exchange) and MTLSD (Multi Time Location Storage and Diffusion) clone detection methods have been discussed in [12]. Accordingly, the concept of swapping time position is claimed in the MWSN communication area of every encountered nodes. In Static WSN, the authors [13,14] proposed the clone detection solution using cluster based. Localization based detection approach has been explained in [15].

## 3. Proposed Security Model

### 3.1. Network Threat Model

The network model is assumed as homogeneous consisting of *N* quantity sensor nodes in the considered DT technique. In the proposed approach, it is assumed that the random way point model for every single mobile node movement though their position and location would be different with neighbor nodes at T time period. In a fixed battery scenario, to perform a competent process such as sensing data and exchanging information in MWSN, the system needs to replace dead nodes with new mobile nodes. Moreover, it is considered that every mobile sensing node can be identified through cluster head via communicating and exchanging data (i.e., battery, key, location, ID) in the form of packets to each cluster head. A distinctive key and identity will be assigned to every mobile node earlier to deployment by network administrator and the availability of bidirectional communication links between mobile nodes is also considered. It is noted that every mobile node is capable to know its location via localization method [16,17]. The clone threats can occur through advisory actively or passively. Passive Attacks are those in which an advisory just takes the data of the original mobile node. Active attacks are capturing and stealing all the data in addition to inserting dangerous attacks in the network. Clearly, the active attacks are more harmful and malicious. An advisory can generate numerous clones of the same mobile node. In the proposed method, we suppose that advisory cannot change the key of the nodes although it has capability of fully controlling mobile nodes. In this scenario, the attacker can use the captured nodes to perform dangerous situations, as both nodes (original and clone node) have same features.

### 3.2. Cluster Assumption

The mobile nodes are generally deployed in clusters [18] where each cluster has a cluster head (CH) [19]. In MWSNs, the operation of replica detection relies on the performance of cluster head, as cluster head is responsible to transmit data, communicate with other cluster head and arrange the information before transmission. Therefore, it is essential to pick a mobile node that has the capability to achieve the above target [20]. In this paper, the following features are assumed for the deployment of cluster:

1. The maximum battery level should necessitate for cluster head to accomplish the responsibilities.
2. The high-level connectivity and coverage must have a chosen cluster head in MWSN.
3. To abate the transmission time, the selected cluster head should be placed near the BS (Base Station).
4. The cluster head is fixed over time for the predefined detection period.

**Figure 1:**
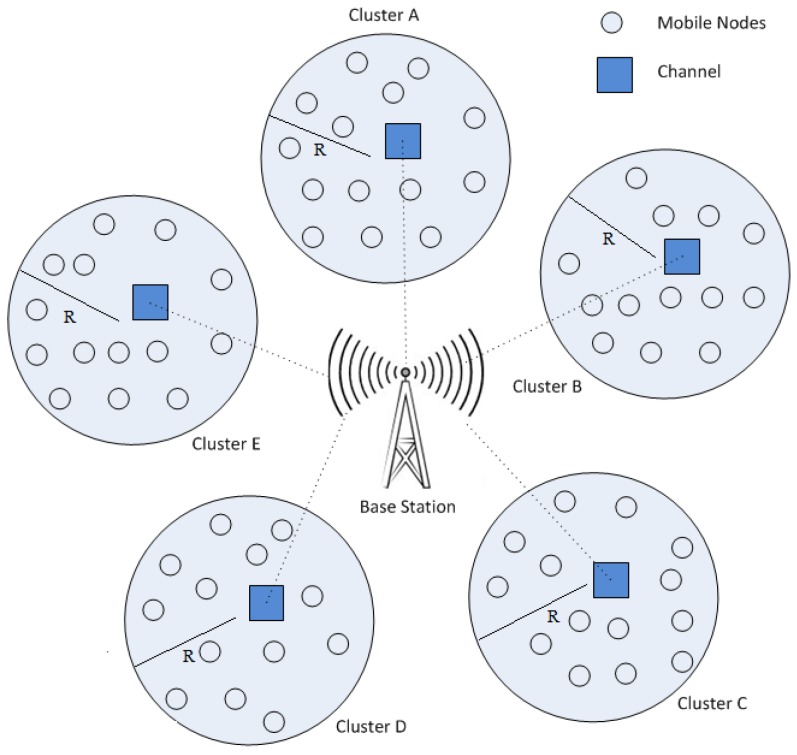
Cluster in MWSN.

Figure 1 shows the clusters in MWSN where they are grouped together in circles with radius R. Basically, clustering algorithm is mostly used to enhance the network’s life time in MWSN. It carries out self-organizing and re-clustering operations for cluster head selection in each detection period. Accordingly, one mobile node performs the tasks of CH and other mobile nodes take action as the member of the clusters. CH communicates with members of the mobile nodes and sends the collected information to the BS. It is dependent on two stages: First is the setup stage and second is the steady state stage. Formation of clustering and CH occurs in the first phase. Mobile nodes elect the cluster head independently. The chosen CH transmissions the information via Carrier Sense Multiple Access with Collision Avoidance Medium Access Control (CSMA/CA MAC) protocols [21]. Associate mobile nodes choose the cluster head (i.e., CH) by computing Received Signal Strength Indication (RSSI). After the CH captures the information and connects to the cluster, the cluster head (i.e., CH) creates a Time Division Multiple Access (TDMA) found list for its members and allots a time period slot for transmission for every node. The second stage is initiated after selecting the cluster and CH in MWSNs. In steady state, mobile nodes establish connection to CH through allots slots depending upon the period. Otherwise, mobile nodes lie in sleeping state. Once CH receives information from all associated mobile nodes, it would start the data transmission towards the BS [22].

## 4. Danger Theory (DT) Based Approach

**Figure 2:**
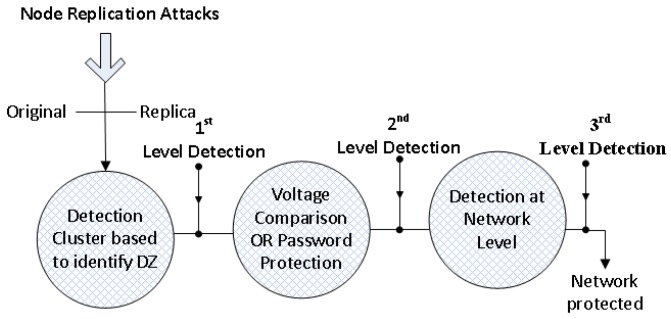
Clone Detection Stages in MWSN.

The DT idea was introduced by Matzinger [23] and Mark Burgess [24] further explained the danger theory concept in 1998 in computer systems for protection. We introduce the DT method for clone detection by involving the multiple stages. Figure 2 demonstrates the various steps to detect and mitigate the clone process via DT mechanism. The DT approach introduces three levels of the detection and mitigation process. The first step is to highlight the abnormality of DZ in mobile nodes that is fishy. This factor for clone detection can be seen in terms of true positive and false positive. The second step depends upon battery check and random number password, if two mobile nodes are found with the same ID and different key (battery check) and if an identical ID is found with an identical key (random number-based check). The third stage is for securing MWSN, the key role is to update all about the clone node.

### 4.1. First Level Detection

If two or more replica mobile nodes with the same ID and features are deployed, it would indicate the presence of a clone mobile node. The replica mobile node will involve stealing the communication as an advisory which can lead the information to be misused actively in MWSN. Moreover, he/she could control and monitor the system. Hence, the cluster-based approach is introduced to monitor the abnormality in the mobile nodes. Every mobile node exchanges the information to the CH in the packets form. Each CH monitors the frequency of mobile node meeting (i.e., (λ)), monitor that uncertainty if lambda is beyond the set range; it alarms the clone’s existence in MWSN. Furthermore, the identified cluster known as DZ is established by the mobile node location in a minimum time period. In Algorithm 1, the frequency of the identical node meeting is achieved through checking the predefined threshold. The CH will generate a message to BS, to highlight the DZ (Danger Zone) as illustrated in the algorithm.
**Algorithm 1** Cluster Based (First Level Detection)**Input:** Check whether the frequency of node meeting (λ) is greater than or equal to the threshold value (ζ).**Output:** Identification of replica(s) in MWSN by highlighting the Danger Zone.**Description:**1:Initialize mobile nodes;   2:**for** (*i* = 0; i≥ζ;i++);3:Cluster header elect based on LEACH protocol4:Broadcast the message to all neighbors nodes in cluster transmission range of the cluster head   5:**If**λ≥ζ then;6:Abnormality in network7:Broadcast message to BS8:Cluster←DangerZone (highlighted by BS)9:Return 0;   10:**Otherwise**11:Normal12:Return 1;13:Return 2;   14:End

#### 4.1.1. Data Exchange between Mobile Nodes

**Figure 3:**
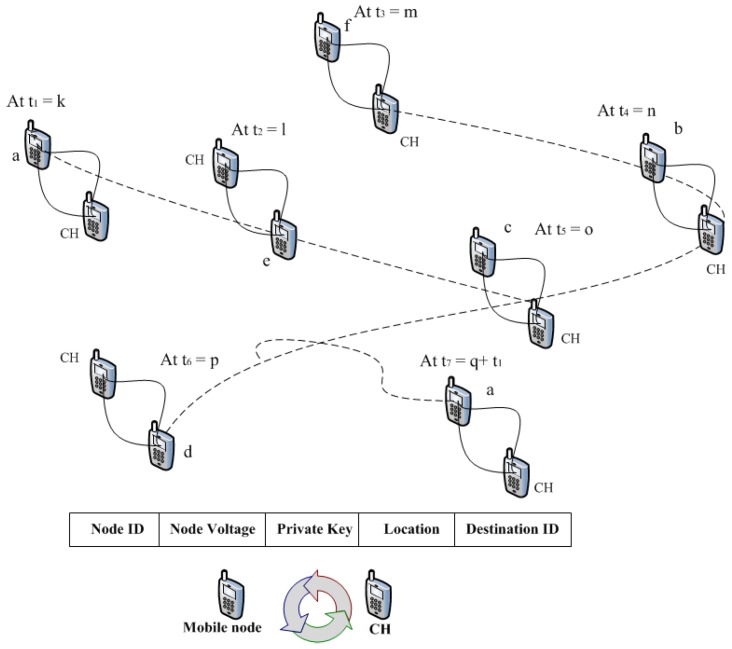
Mobile node encounter to other node at various time intervals.

For each precise detection interval, a mobile node at certain time varies its location and interconnects with the near mobile nodes [25], as shown in Figure 3. Every mobile node when encountering mobile node CH (i.e., a and b node meeting) in the specific period, the data is stored in their memory for exchanging information.

$$\begin{array}{cc} \; & {\mathrm{Message}}_{a\to CH}:(a||V_{a}||ID_{a}\left|\right|k_{a}\left|\right|L_{a}\left|\right|D_{a})\\ \; & {\mathrm{Message}}_{b\to CH}:(b||V_{b}||ID_{b}\left|\right|k_{b}\left|\right|L_{b}\left|\right|D_{b}) \end{array}$$

where $ID_{a}$, $V_{a}$, $K_{a}$, $L_{a}$, $D_{a}$, $ID_{b}$, $V_{b}$, $K_{b}$, $L_{b}$, and $D_{b}$ are the identities, voltages, private keys, locations and destination IDs of node *a* and node *b* respectively. Figure 3 explains the entire sequence i.e., movement of mobile node, the changing of its position at different time intervals and the exchange of information based on packets, whereas each packet transfers the information based on the node identity, voltage, key, location and destination identity [26]. The concept of node information exchange is explained as follows: Let *a*, *b*, *c*, *d*, …, denote the mobile nodes in the MWSN and $t=t_{1}$, $t_{2}$, $t_{3}$, $t_{4}$, …, $t_{n},$ equal time interval and associate the time interval with the cluster head meeting (i.e., CH). Once the random mobile node (i.e., *a*, *b*, *c*, …) meets the CH node, the CH node computes a time *t* for each node meeting. Specifically, using *k*, *l*, *m*, *n*, *o*, *p*, *q*, ……, to explain the time of node meeting, where $t_{1}<t_{2}<t_{3}$ given k<l<m. Assuming *a* is a mobile node and it meets CH (cluster head) at time $t_{1}$, where k<l, CH meets the mobile node *a* again at time $t_{7}$. Thus, CH meets twice with mobile node *a* at a distinct period, as shown in the the above figure, and this indicates the similar node IDs (i.e., *a*) meet with cluster head.

#### 4.1.2. Frequency of Mobile Node Meeting

The clone threats can be traced via the frequency that the mobile nodes meet each other. The action of the mobile nodes are distributed into two groups: (i) Ordinary scenario *A* (i.e., no clone node) and (ii) anomalous scenario $A^{*}$ (i.e., existence of clone).

(1) $$\begin{array}{c} Z\left(t\right)=\left\{\begin{array}{cc} \mathrm{Normal}\;(A) & \;\mathrm{if}\;\lambda <\zeta \\ \mathrm{Abnormal}\;(A^{*}) & \;\mathrm{if}\;\lambda \geq \zeta \end{array}\right. \end{array}$$

where lambda(λ) characterizes the mobile node meeting (i.e., frequency) and random threshold stetted value represented by Zeta (ζ). The mathematical expression demonstrates that there could be a chance of the presence of a clone when the stetted threshold is beyond the frequency of the mobile node meeting. The equation indicates A(i) as the normal situation, while $A^{*}\left(i\right)$ as the abnormality (i.e., clone mobile node), that comprises the entire *i*th counters. Consider the detection probability rate in the form of Z(i) as below,

(2) $$P_{r}\left(A^{*}\left(i\right)\right)=\sum_{i=1}^{k}Z(i)$$

Let λ, for clone detection depend upon mobile node meeting frequency can be expressed as,

(3) $$\begin{array}{c} \lambda =\left\{\begin{array}{cc} \lambda_{S}=P\left(R=1|A^{*}\right) & \mathrm{if}\;\mathrm{IDs}\;\mathrm{with}\;\mathrm{same}\;\mathrm{key}\\ \lambda_{D}=P\left(R=0|A^{*}\right) & \mathrm{if}\;\mathrm{IDs}\;\mathrm{with}\;\mathrm{different}\;\mathrm{key} \end{array}\right. \end{array}$$

where the clone existence conditional probability is either the similar identity with similar private key is $P(R=1|A^{*})$ and similar identity and different key in MWSNs can be defined as $P(R=0|A^{*})$. Hence, the replica mobile node can be expressed by:

(4) $$P\left(R=1|A^{*}:\mu_{i}\right)=\sum_{i=1}^{k}Z(i)$$

The number of counters cab is shown by $\mu_{i}$ while *i* represents the value from 1 to *k*. By solving this expression and extracting the sum of conditional probability by,

(5) $$\begin{array}{c} 1-\prod_{i=1}^{k}(1-P\left(R=1|\mu_{i}\right)\;\mathrm{if}\;R=1 \end{array}$$

(6) $$\begin{array}{c} \prod_{i=1}^{k}(1-P\left(R=1|\mu_{i}\right)\;\mathrm{if}\;R=0 \end{array}$$

By computing the above mathematical expression (5) and (6), the clone detection probability can be calculated as below,

(7) $$\lambda_{S}=P(R=1|A^{*}:\mu_{i})=1-\prod_{i=1}^{k}(1-P\left(R=1|:\mu_{i}\right)$$

(8) $$\lambda_{D}=P(R=0|A^{*}:\mu_{i})=\prod_{i=1}^{k}(1-P\left(R=1|:\mu_{i}\right)$$

λ≥ζ represents the clone node presence in the MWSN, where ζ represents the threshold analyzed by the network operator.

#### 4.1.3. Setup of DZ (Danger Zone)

**Figure 4:**
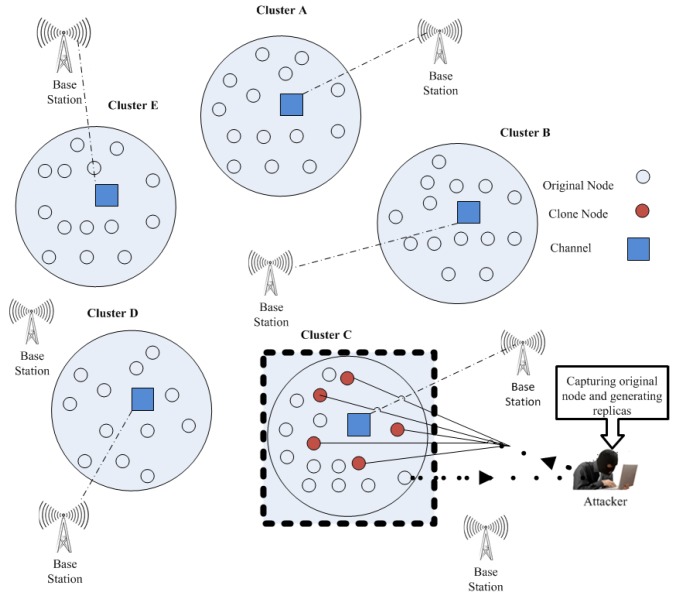
Cluster level detection in a Mobile Wireless Sensor Network (MWSN).

In DT approach, the unique concept term DZ is used to identify the clone. Basically, the concept is to highlight the infected area where an attacker can insert clone mobile nodes. The idea of DZ would be helpful for fast detection of replication attacks. By using this concept, it can focus only on infected areas instead of the entire network for replication attacks detection. Consequently, the CH will inform the network (i.e., BS) about the clone presence in MWSN and afterwards will indicate the affected cluster by the mean of DZ. Figure 4 explains the ordinary scenario where it later warns about the clone mobile node presence and, highlights the area of specific cluster by means of danger zone. Supposedly, the cluster area remains the same before and after deployment, although the mobile nodes are in motion. Furthermore, if the *T* detection interval is in MWSN, considering that the motion model for mobile node is the random way point mobility model, then the possible clone existence in the specific cluster can be signified as DZ.

### 4.2. Second Level Detection

The first stage of detection is completed after the highlighted infected area (i.e., DZ) and then the second stage of detection is initiated. In the proposed DT approach, two scenarios have been raised with the solutions: First is based on battery comparison of two nodes and the other is random number based. In battery comparison, checking the voltage level as the clone mobile node is deployed after the original. Hence, it should have maximum voltage to capture and monitor the network [27]. Moreover, replica will prerequisite to have higher levels of battery to control, observe, interconnect and screen various tasks. In random number method, the CH will enquire the indicated mobile node to exchange numbers (i.e., password). The right number will confirm the original mobile node, otherwise it would be located as a clone. Algorithm 2 describes the whole procedure of clone node in detail. This idea has been raised to increase the detection rate at the first stage of identification but there are also several chances that the original mobile node encountered CH many times in the same time interval. Therefore, the second stage detection is indeed important in order to differentiate between the clone and original mobile node to suppose the statistic that if same identity and same key, other same identity with a different key. To sort out this problem, two types of strategies have been considered, which are:

1. Battery Check
2. Random Number

**Algorithm 2** Battery Check and Random Number (Second Level Detection)**Input:** Check the battery or random number of nodes claiming the same ID. In the case of voltage level comparison, the nodes that have greater voltage would be a replica else performed the task based on password checking.**Output:** Verification of replica in MWSN by voltage comparison or password of same IDs.**Description:**1:**If** mobile node ID matched with different key;2:Compare the voltages   3:If $V_{j}>V_{i}$4:Replicated node5:Otherwise Original Node   6:**Otherwise** mobile node ID matched with same key;7:Ask Password;   8:If correct password9:Original node   10:Otherwise11:Replicated node12:End

#### 4.2.1. Battery Check

In [28], the authors discussed the various categories of batteries frequently utilized in the mobile nodes, their voltage rate for initial start-up and ending interval. Mobile nodes will encounter the information based on the mobile node ID, battery level and key during the meeting with CH node in a precise time limit [29]. The CH will establish a list for specific intervals in memory and will also build the connection to broadcast the message. Accordingly, the presence of two or multiple mobile nodes with identical ID directs that the clone node exists in MWSN. This conclusion is derived from the assumption that to establish, control or screen the entire system, an adversary requires maximum capacity of voltage and the factor that the clone is deployed after the real node too. Hence, we assume that the clone has a greater value in contrast to real one. Battery types, for example Zinc Manganese Dioxide and LeClanche, are not advised to be used in sensor networks because of their lower capacity and huge charging time as discharging occurs by itself in the storage and it discharges quickly. Some batteries have low cost specification like Alkaline with a high capacity and low discharging, hence it is highly suggested in MWSN. It is much difficult to charge a huge number of mobile nodes in MWSN, which is why the battery used in the network is not rechargeable. The battery-based comparison is established by screening the voltages in the network operation. Furthermore, the battery level rapidly goes down because of discharging abilities and due to rapid change in the discharge rate capacity that varies as well. The author in [26] explained the method to measure the MICA2 sensor node.

**Lemma** **1.** *Comparison based on battery: Consider $B_{i}$ is a Battery level of a real mobile node which is deployed at the initial time (i.e., t = 0) and $B_{j}$ is the battery level of a clone which is deployed after time t in the MWSN (i.e., $t+t^{\prime }$). $B_{j}$ is deployed later at time $t+t^{\prime }$ while $B_{i}$ at t = 0, hence $B_{j}$ has a greater value. Furthermore, to control and screen the information advisory requires a huge voltage level, else will fail the aim to produce clone. Besides, if $B_{i}\neq B_{j}$*

*At t=0; $B_{i}\approx B_{max}$*

*At $t=t+t^{\prime }$; $B_{i}\approx B_{max}-B_{consumed}$*

$B_{j}\approx B_{max}$

$B_{i}<B_{j}$

This demonstrates the expression for mobile node deployment with specific time and clone comes after $t+t^{\prime }$ of real mobile node, hence has a maximum battery capacity. With this consideration, it has a probability of high false positive and low true positive rate. The proposed DT based method is computed for maximum efficiency in terms of true positive and false positive rate by contrasting the battery level.

#### 4.2.2. Random Number Based

As discussed in the literature review section, the XED (extremely efficient detection) [30] is built with the logic of exchanging the random numbers at various locations. The approach is based on the concept of remember and challenge. Accordingly, uncertainty did not encounter the correct number or the value were not the same (based on random number), which indicates the existence of a clone. By using this concept in the DT approach, replication attack can be reduced (replica meets the cluster head again at a different time interval, if frequency of node meeting with similar IDs and same private key (i.e., $\lambda_{S}$)) occurrence in MWSNs. The CH transfers the information to the identified node (similar IDs with similar key features) to encounter random value. It would be a clone mobile if it encounters false information. If the CH encounters to the mobile node at t time interval $\lambda_{S}$, *r* is random number value and *S* is IDs of identical mobile node. Accordingly, CH will send the message to the ’S’ (identified same IDs) to encounter a random number. The check depends upon the binary numbers, if r=n indicates that the exchange number is wrong, and then the clone mobile node is identified. Conversely, if r=r, an identified node is the original one. The method to check the value, if the right (i.e., r=r→R) is provided, it would be the original node. Otherwise, it will be considered as a replicated node (i.e., r=n→W) in MWSN. The password check procedure will only be initiated if the mobile node has similar IDs with similar keys. Furthermore, memory overhead has been calculated to show the minimum memory utilization in the proposed DT approach. Therefore, O(1) is the calculated value of the memory overhead, which permits the count to be constant and independent of the many mobile nodes meeting in the verification process of the replicated node. The memory is adequate to collect the data since a precise number of mobile nodes are executing this job. Thus, the CH maintains the constancy of the number check as it finds identical node identity with identical key by means of memory cost O(1). A distinct approach to check random number with identical identity mobile node has the ability to detect replica. This quick detection is the main feature of the proposed method which distinguishes itself from other existing clone detection approaches.

(9) $$\begin{array}{c} s=\left\{\begin{array}{cc} \mathrm{Replicated}\;\mathrm{node} & \;\mathrm{if}\;r=n\\ \mathrm{Original}\;\mathrm{node} & \;\mathrm{if}\;r=r \end{array}\right. \end{array}$$

### 4.3. Third Level Detection

**Figure 5:**
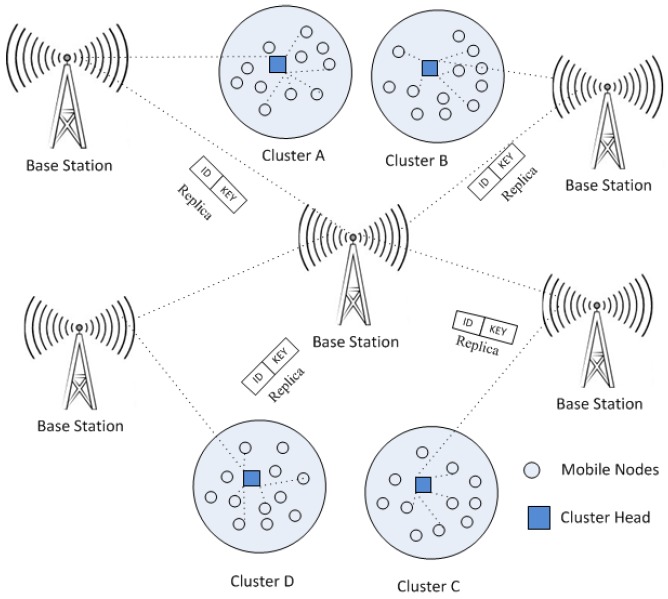
Secure network by broadcasting message about clone.

The third stage will start after the confirmation of the clone mobile node presence. Most of the existing proposed methods to detect clones are considered the detection of clone rather than securing the entire system. To sort out this issue, third stage is proposed. So, the CH will transmit the information about clone mobile node to the BS. The inspiration to initiate the third level detection is to highlight the mitigation process, as many of the existing approaches only focus on detection instead of prevention of replication in MWSNs. Figure 5 proves the task of network protection through broadcast information about clone to other BSs. It should be noted that by broadcasting IDs of clone node, it would be difficult to differentiate between the clone and the original one. Therefore, it is necessary to transmit the communication to the entire BSs about clone presence (i.e., specific node Identity). The entire process of MWSN protection is explained in detail in Algorithm 3, as there is probability that the clone will move into near clusters and comprise himself another time in dangerous threats. To prevent the replication attacks, this approach broadcasts the information about clone to another cluster through BS. Thus, by adopting this approach, the MWSN can be secured from malicious threats vastly.
**Algorithm 3** Network Protection (Third Level Detection)**Input:** To inform other BSs about replica through the replica node’s ID and Key.**Output:** Protection from replication attacks in MWSN through replica node’s ID and key.**Description:**1:Node Replica;   2:**If** Broadcast the message to other BS through ID and key.3:Networks Protected from replica attacks.4:Return 0;   5:**Otherwise** Unprotected Networks;6:Return 1;7:End

## 5. Performance Evaluation

### 5.1. Simulation Parameters

The simulation design for MWSN is composed of N nodes (i.e., 200 for this simulation), size of network is 500 m × 500 m, communication area is 3–25 m, randomly deployed, mobility model is RWPM, clone varies in range between 10 to 70, Base Station (BS) is (150,195), alkaline battery, time for is 300 s, clusters number is 3 to 10 depending on the design, bit rate is 9600 bps, speed is 3 m/s, size of packet is 24 bits.

### 5.2. Simulation Results: Different Parameters’ Effects on Replication Attack Detection

The DT method evaluates key factors recognized as true positive, false positive and false negative. The DT based approach involves three major security parameters; true positive, false positive and false negative. Furthermore, it also shows the impact of other performance parameters such as detection accuracy, communication overhead, memory overhead, detection rate, energy, effect of lambda, network life time, detection time, delay impact and comparison of SPRT (centralized approach) and XED (distributed approach) in detail. The outputs show the average value for 20 simulation runs, and it uses the average or stabilized value to analyze the replication attacks performance. The parameters used for detection of clones to evaluate the hybrid process are defined and discussed as follows.

#### 5.2.1. Detection Accuracy

**Figure 6:**
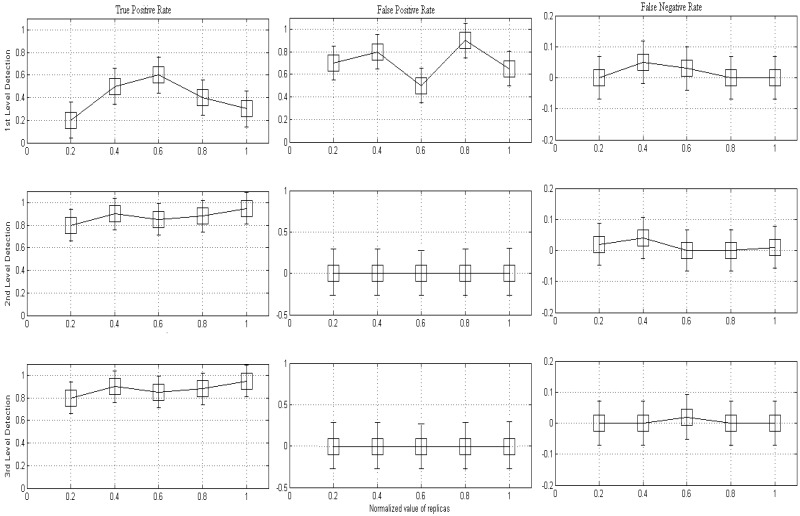
Different stages of clone detection in MWSN.

The DT method evaluates key factors recognized as true positive and false positive. The effectively detected clone is identified as true positive, however, unsuccessfully rate is known as the false positive. The simulation execution of the proposed danger theory at multiple stage is illustrated in Figure 6. In the figure, the high accumulation rate of clone identification is at a distinct phase. It can also be observed that at each stage, the true positive rises. As the proposed method consists of multiple stages in mean to detect the clones, the first stage is designed for verification, in addition of the identification of the anomaly at cluster to establish the danger area (i.e., DZ). The second stage is towards mitigating the clone threat by contrasting the battery level and encountering the random number check while the third stage is to prevent the MWSN from dangerous attacks by transmitting messages through BS to others. To categorize the sheltered network from clone attacks, there is a framework analysis to improve the true positive (i.e., successfully replica detection) and decrease the false positive rate (i.e., wrongly detected replicas). Therefore, the proposed DT (multilevel detection) method has an impact on the development in clones’ threats detection execution.

It can be noticed that the first stage detection screens minor clone detection rate (i.e., in term of true positive), below 0.6 besides vast false positive rate, higher than 0.5. However, in the second and third stage, it validates the equivalent behavior of true positive rate that is approximately 0.95. Besides, the false positive percentage in the third stage is reduced to zero as likened with the first and second stage. The multiple stages simplify the clone detection application in terms of true positive. In relation to clone threats, it can be observed that a multilevel clone revealing is additionally proficient and competent. Moreover, the multi-level clone detection also confirms the minimum rate of false positive and negative. In the figure, it can be observed that a fewer percentage of 0.1% is for false negative.

The cause of this outcome is that there is a probability that the CH does not encounter again through mobile node first stage of clone detection, which will increase the rate for false negative. Moreover, due to the mobility features in nodes, in the detection period, there is the risk that some may travel outer side of the specific area (i.e., cluster). Since the clone exists later than the real mobile node, it is obvious that the battery level of the clone must be higher than the genuine mobile node. Additionally, in the least time interval, the DT can identify the clone or fake mobile node in the exact cluster area. In this way, an attacker cannot take advantage of spreading clones in MWSN. The execution outcomes display that the DT technique is fast, competent, secure and intelligent for clone detection. A comparison with existing (centralized and distributed) approaches is discussed in Section 4. The DT approach is restricted with static battery types for mobile nodes. In the situation of rechargeable battery or solar cells, the clone recognition may be very tough and challenging with respect to their battery level.

#### 5.2.2. Communication Overhead

**Figure 7:**
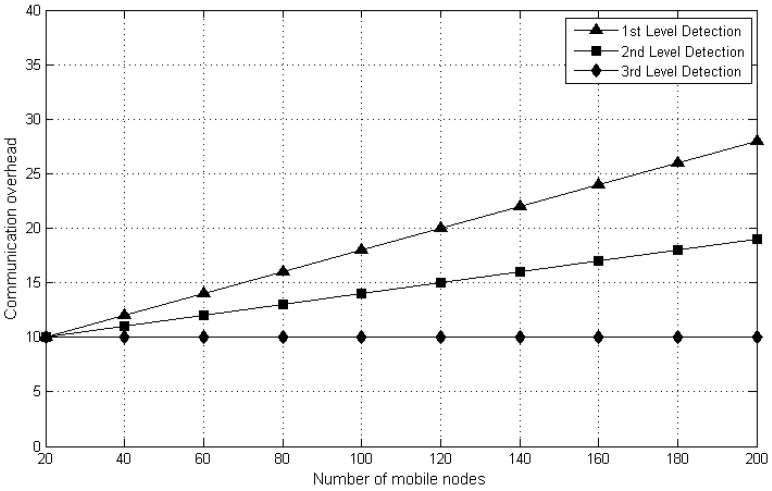
Communication overhead of Danger Theory (DT) approach.

The total number of messages forwarded via mobile nodes in MWSN is described as Communication overhead. If mobile nodes (i.e., *N*) are receiving and delivering the data via messages and every single message must transfer by CH, 1 × *N* would be the overall communication overhead in MWSN. For the proposed DT approach, it is O(N). Figure 7 screens that the overhead used for the proposed DT approach is lesser than that of other approaches. This is because the danger theory concept is built on cluster or cloud and every single mobile node must transfer the data to CH. Besides, O(N) depends upon the entirely used mobile nodes with direct proportion. Hence, if there is a rise in the quantity of mobile nodes, then communication overhead would also be increased. The result shows that the first level and second level of detection communication overhead increases with the number of mobile nodes. However, third level detection does not show salient changes because its task is to only forward the message about replica to the other BSs.

#### 5.2.3. Memory Overhead

**Figure 8:**
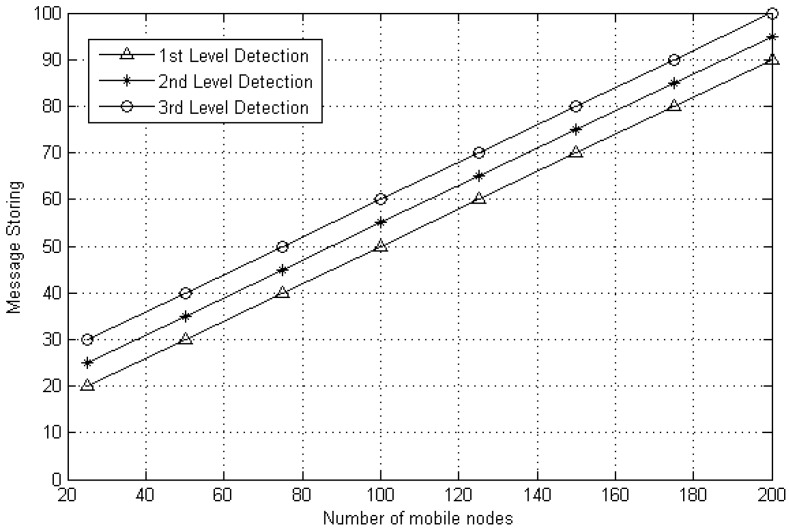
Message storing of DT approach.

Message storing expresses the whole messages consumed in MWSN for replication attacks. In Figure 8, it can be noticed that the proposed method has a maximum number of messages stored. In execution outcome, the third stage of clone detection presents a maximum storage which is 10% and 20% compared to the second and first stage. It should be noted that the message storing reflected in the third level detection is the sum of both first and second stage respectively. Therefore, the third stage has a maximum number of messages stored. Each mobile node must save the data, verify it, make a contrast that is based on similar identity. Thus, for the clone identification process, every single mobile node must consume mobile node memory to encounter and save the packets that is based on ID, key, location, exchange on random number, etc. The CH requires to store simply one message per mobile node, and for this reason, only *N* messages are required to be stored at cluster head.

**Figure 9:**
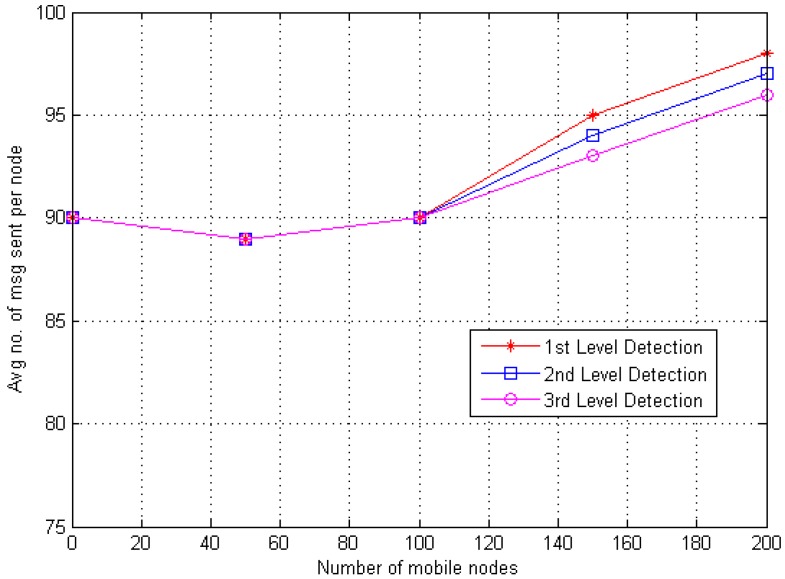
Average amount of messages transmitted per mobile node.

**Figure 10:**
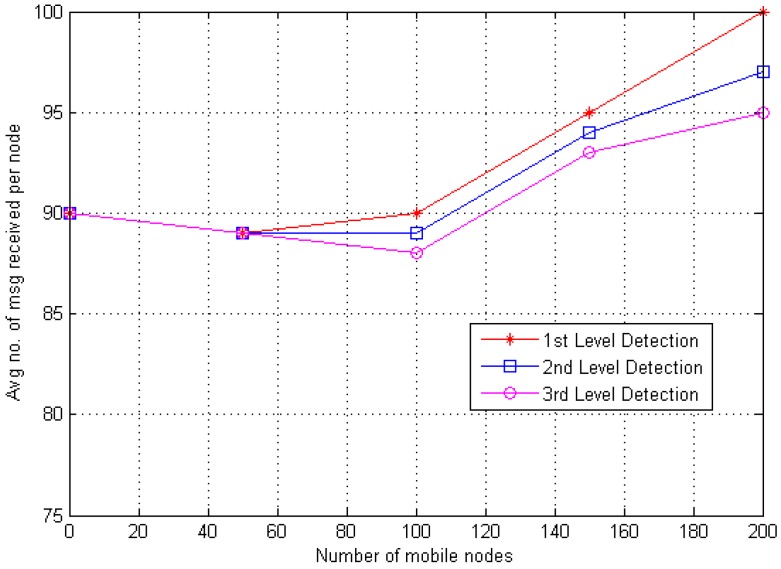
Average amount of messages delivered per mobile node.

Every CH stores the information of all communicating (i.e., *N*) nodes, and every CH has an individual queue of the same dimension. Hence, the memory overhead for battery level check can be defined as O(N) and random number based is O(1) (i.e., 1 represents the fixed number of counts) as mentioned earlier, therefore the estimated memory overhead O(N) is for DT approach. Figure 9 and Figure 10 explain the encounter information (i.e., sent or received messages) for every single node. Hence, results (i.e., first stage, second stage and third stage) show that the average number of messages sent and received increased as the quantity of mobile nodes increased.

### 5.3. Energy

For clone detection using the DT approach, another important factor is energy as an advisory needs high power to control and monitor the MWSN.

**Figure 11:**
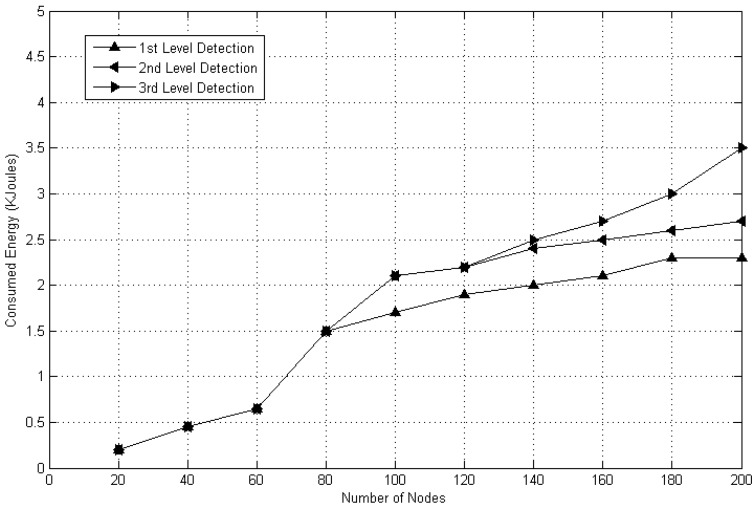
Energy consumption at different stages of the DT method.

Correspondingly, to accomplish a better performance for clone detection in MWSN, the energy level must also be considered. Figure 11 displays the level of energy consumed by using DT concept at multiple phases of detection. It shows that the energy consumption values at first, second and third level detection among 80 mobile nodes are similar. It is also observed that by utilizing fixed batteries types, the third level detection consumes more energy than the first and second level detection.

### 5.4. Network Lifetime

**Figure 12:**
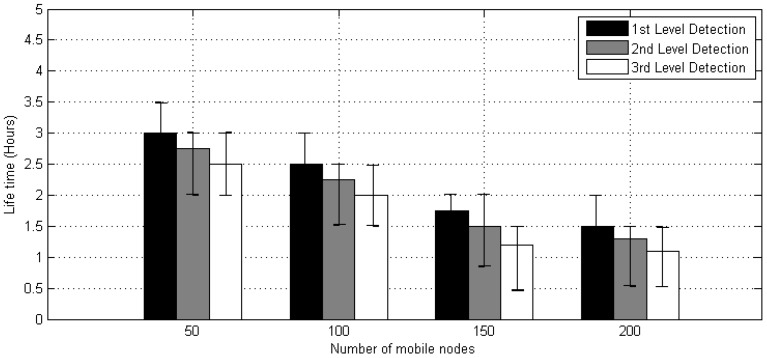
Lifetime of nodes.

The DT method also illustrates the multilevel (i.e., first stage, second stage and third level stage) performance term of network lifetime. It is a time period when mobile nodes are deployed till the first mobile nodes die because of the usage of battery. In the MWSN, the amount of the mobile nodes is enhanced and varied between 50, 100, 150 and 200. Figure 12 explains that the network lifetime is stable for 50 nodes. However, as the number of the mobile nodes increases, the lifetime of network decreases due to the usage of fixed batteries.

### 5.5. Detection Time at Different Levels of DT Approach

**Figure 13:**
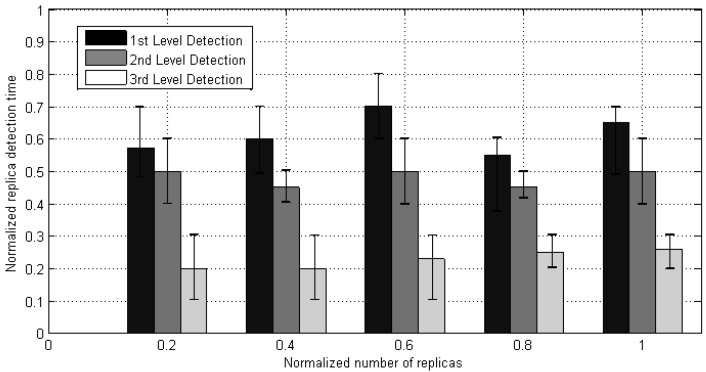
Detection time.

Figure 13 characterizes the stabilized recognition time of clone threats in network at multilevel detection.

Based on the experimental results, we noticed that the detection period of the first stage depends upon the lambda (i.e., frequency of node meeting). Besides, detection time will be increased if they do not encounter another time. The second stage clone detection interval is smaller than the first one because it depends upon the battery level or random number check as mentioned in Section 3. Meanwhile, the third detection stage contains the least period as only depends on exchanging the clone messages based on identity and key, then broadcasting the information. The fluctuation in Figure 13 shows the different detection intervals in MWSN (i.e., the nodes are mobile and clone detection is dependent on the nodes meet each other).

### 5.6. Delay Impact on Detection Efficiency

The crucial task is to control the time in terms of clone detection delay in the proposed multi-stage detection method. Parameter “*d*” (i.e., time out phase for receiving message) involves checking the delay in clone detection.

**Figure 14:**
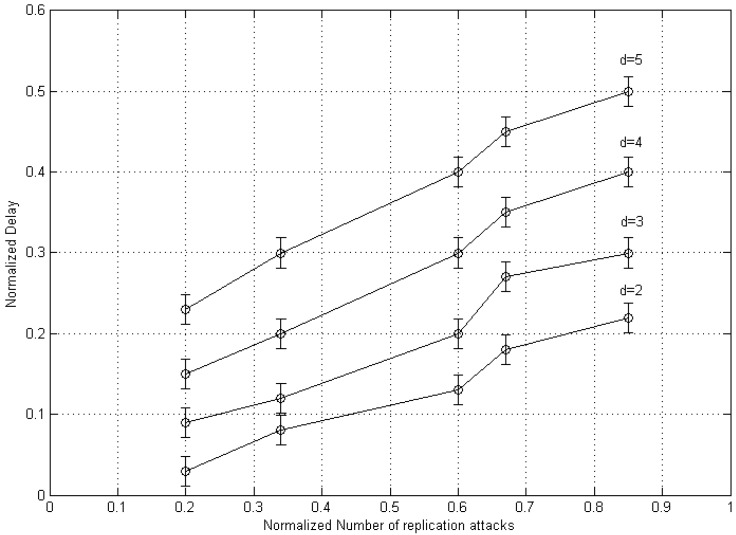
Delay effect of DT approach on detection performance.

As presented in Figure 14, a massive amount of clone mobile nodes is placed in networks, therefore the rise in the value of *d* will disturb the performance of the mobile nodes because of time delay. Results show that both are directly proportional to each other. Hence, detection delay will increase with rising the factor “*d*” significantly. The mentioned situation should be sorted out with utilizing the suitable clustering approach or by neglecting the nodes that move outside during the detection period.

## 6. Comparison and Discussion

Simulation experiments have been performed to compare the proposed DT based method with SPRT [4,5] (centralized) and XED [30] (distributed). The effectiveness of the existing clone detection approaches, e.g., SPRT and XED depend upon fast detection and simple remember and challenge response (i.e., based on random number), respectively. Therefore, it motivates this research to compare the proposed DT approach with SPRT and XED in order to show the efficiency (i.e., clone detection improvement) to secure the MWSN from malicious activities.

The fair comparison is performed by using the same estimation, simulation parameters and network settings. Previous research [31] has compared SPRT (i.e., centralized method) and XED (i.e., distributed method) by using specific simulation parameters. Therefore, this paper has used the same consideration and estimation to perform a fair comparison between SPRT (i.e., centralized method), XED (i.e., distributed method) and hybrid (i.e., DT) approaches. The comparison is made consuming several execution factors, namely, replica detection ratio, completion time for clone identification, energy consumption, false negative and false positive for replication threats. The details of the parameters can be described as follows:

1. **Energy Consumption for DT ($E_{d}$):** This is the total consumption cost in support to communication and computation overhead for clone detection.
2. **Clone detection ratio ($P_{d}$):** This is the ratio of successful clone identification interval over the whole experiments’ execution.
3. **Completion time for detecting a clone ($T_{d}$):** This is the interval that is consumed when the first clone detection happens.
4. **Errors for False Positive:** This indicates the wrongly detected replicas in MWSN (a normal node is detected as a replica).
5. **Error for False Negative:** This reflects that clone mobile node is not identified as a clone, which may collapse and affect the capability and performance of clone detection due to wrong identification.

These parameters will briefly reflect how centralized and distributed methods for clone identification is effective and more efficient in network. The single point of failure is the main concern in the centralized approach as mentioned in [32] that affects the tasks in MWSN. Moreover, the mobile nodes deployed close to the base station could suffer from the huge load which would eventually lead to congestion (i.e., load balancing issue). That could be in favor of an advisory. Therefore, for clone identification, a balanced and distributed technique is obligatory to overcome the drawback of the single point of failure.

For clone identification with distributed method, as observed in [30], it mostly has issues with memory, for example XED if mobile node memory is fully occupied, for localization and detection time completion. On the other hand, it has less load and output shows that it has least errors in terms of false negative and positive. In order to decrease the matter of memory, hybrid method (i.e., centralized and distributed) is used for clone identification and mitigation. By mean of fair comparison, we utilized the same simulation parameters approximation as mentioned in [31]. Accordingly, the performance evaluation involves 200 mobile nodes, network size of 500 m × 500 m, transmission range of 100 m, detection time of 1000 seconds and average simulation results of 100 simulation experiments.

**Figure 15:**
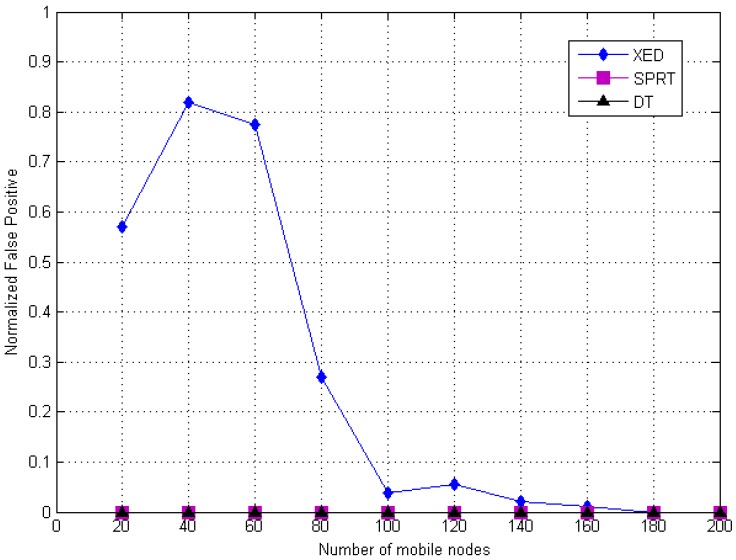
Comparison of simulated value of false positive rate of the DT (hybrid) detection with SPRT and XED.

**Figure 16:**
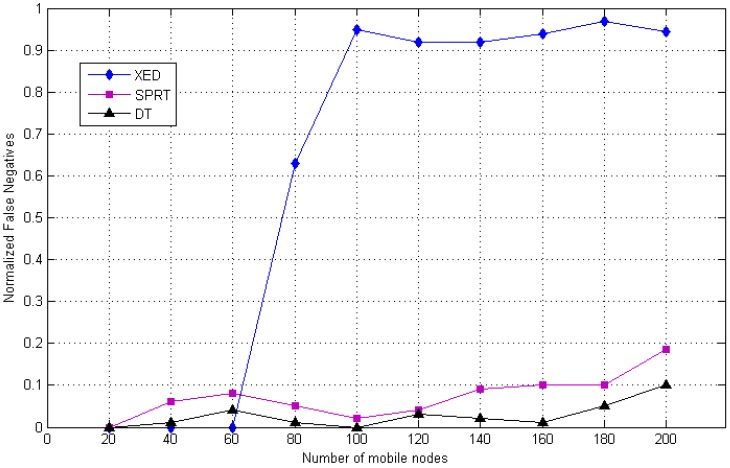
Comparison of simulated value of false negative rate of the DT (hybrid) detection with SPRT and XED.

Figure 15 and Figure 16 illustrate the false detection errors of the clone detection methods in terms of false negative and false positive, respectively. It could be seen that the multi-level DT based clone identification approach offers efficient outcome compared to XED and SPRT methods. Specifically, Figure 15 illustrates that the proposed DT method and SPRT consume similar false positive rate which improves the replica recognition competence compared to the distributed method (i.e., XED), However, Figure 16 shows that the proposed DT method has a good performance in terms of false negative rates of XED and SPRT.

The false positive and false negative values which are shown in Figure 15 and Figure 16, respectively, highlight the performance among distributed (i.e., XED), centralized (i.e., SPRT) and hybrid (i.e., DT) approaches. Moreover, the calculated average values of the false positive and negative rates for these approaches (i.e., XED, SPRT and DT) are compared based on wrongly replica detection (i.e., false positive rate) and in the situation where replica is still present after performing the detection procedure (i.e., false negative rate). It should be noted that by using the DT approach, there is a false positive rate enhancement compared to XED and SPRT. Figure 15 demonstrates that the performance of the DT and SPRT is same in terms of false positive rate (i.e., clone wrongly detected).

Figure 16 illustrates that the proposed DT approach performance increased in terms of false negative rate compared to XED and SPRT. The results show that the proposed method based on danger theory is efficient, competent and sheltered for clone identification. Generally, as specified in the above figures, it can be concluded that the hybrid, centralized and distributed techniques for clone recognition have a positive impact in terms of false negative. The multi-level method depends on the first stage (identify the infected area), the second stage (battery level check and exchange random number) and the third stage (to secure network).

**Figure 17:**
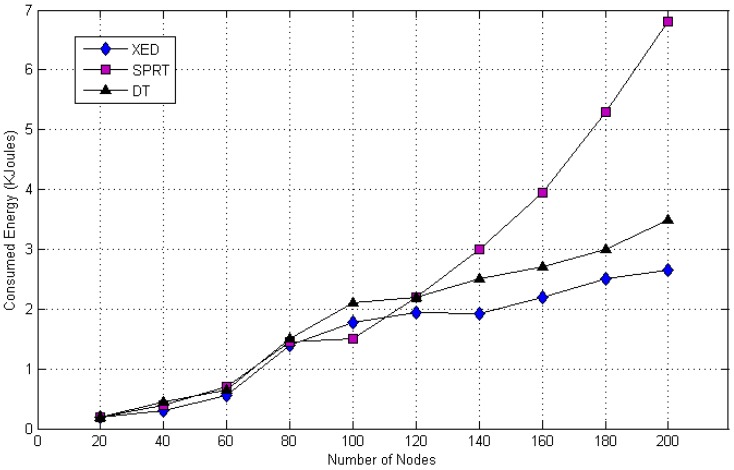
Comparison in terms of energy consumption.

Figure 17 represents the contrast with respect to energy consumption in distributed, centralized and hybrid approaches for clone recognition. XED and DT utilize less energy while SPRT entails higher. The clone detection of the proposed DT and SPRT procedures decrease gradually, whereas the XED method decreases rapidly for the reason of detection errors in terms of false positive and negative. The energy consumption (i.e., $E_{d}$), replica detection ratio (i.e., $P_{d}$) and detection time (i.e., $T_{d}$) of XED, SPRT and DT approaches are tabulated in Table 1. There is a significant improvement for the DT approach as presented in Figure 17 in comparison with the other methods for replication attacks detection.

The SPRT strategy is centralized and may suffer from only one designation collapse (i.e., BS). With this breakdown, the whole network could be affected. Moreover, it has the issues of energy consumption decline and absence in MWSN across one point such as BS. In addition, the SPRT approach is a fast clone detection but it is created by comparing the speed level of each mobile node through adjusted threshold value, hence, it needs precise measurement equipment that could be costly and unaffordable. Furthermore, in the case of intelligent attackers, he/she could adjust the speed level (i.e., which lies in the range of the threshold). The distributed methods can be considered in MWSN to reduce the chance of one-point failure. By studying numerous distributed approaches, it could be observed that most methods depend on the frequency of mobile nodes meeting at a precise time interval. Encountering the random number-based method (i.e., XED), is not considered as a fast clone detection because it depends on the time or frequency of the mobile node meeting. Hence, if two or multiple mobile nodes do not encounter each other at a specific time, it will generate ample delay for clone detection. In the paper [30], clones are not cooperative and do not meet with other mobile nodes, thus clone with no trouble can establish secret communication, so it causes the clone recognition to fail.

Entire solutions for clone detection could come up through distinctive evaluation, inspiration and procedures. Every single method has few merits and demerits aspects. With this basis, it cannot be stated which method is more efficient. Secure communication is a key problem with respect to clone detection. Furthermore, determining clone mobile node scenario obliges quick identification as an advisory could effortlessly compromise, control or screen the MWSN, and later can practice stealing the information. Since clone behaves autonomously and competently in MWSN, there is a chance to encounter several mobile nodes till other loops for detection are initiated and during this period, they can easily capture data like ID, key, secret confidential and so on. Few features can be organized or modified with less effort, for example, with authentication [33,34,35] techniques, false information can be thrown into the network. In MWSN, it is very challenging and motivating to consider about secure environments, considerations and performances for clone threats because of mobility features. To set up an environment with unattended hostile nature is considered very threating and vulnerable, specifically for physical threats identical to clone attacks. Hence, an attacker could generate numerous clones of mobile nodes with identical features. If the clone node is not identified quickly, the whole network will be compromised by an attacker easily and could involve different kinds of threats like screen networks, immoral data aggregation, jam the signals, misuse the data, incorrect data insertion and so on. Thus, clone identification is mandatory and requires abolishing the clone mobile nodes.

## 7. Conclusions

The proposed work projected a DT (Danger Theory) idea for clone detection at different stages. The DT approach basically depends on a multilevel of detection: First stage (which point out the infected cluster (DZ) via examining the frequency of mobile node meeting), second stage (battery level check or random number exchange) then the third stage 3rd (inform about clone to other networks). To show the better performance in clone detection, different factors, specially the true and false positive rate, are deliberated to expose the detection features of the DT approach. It is determined through experiments that the proposed technique attains particularly high true positive, approximately 90%, and less false positive rate. To effectively and competently show the brilliant features of the proposed multilevel method, and to validate the results, the method for clone detection is presented through performing a comparison by assuming the same environment and parameter with existing centralized and distributed methods.

## Figures and Tables

**Table 1:** Node replication attacks performance comparison considering distributed, centralized and hybrid approaches.

# of	XED			SPRT			DT		
Mobile Nodes	$E_{d}$	$P_{d}$	$T_{d}$	$E_{d}$	$P_{d}$	$T_{d}$	$E_{d}$	$P_{d}$	$T_{d}$
20	0.20	1.00	29.740	0.20	1.00	69.610	0.20	1.00	29.740
40	0.30	1.00	45.680	0.40	0.94	117.65	0.45	0.94	30.340
60	0.55	1.00	45.420	0.70	0.92	129.44	0.65	0.98	50.682
80	1.40	0.38	655.29	1.45	0.92	149.91	1.50	0.96	88.79
100	1.77	0.06	950.93	1.50	0.94	106.96	2.10	0.95	107.50
120	1.95	0.08	953.24	2.20	0.94	108.04	2.20	0.95	145.78
140	1.93	0.04	940.30	3.00	0.90	150.92	2.50	0.90	175.43
160	2.20	0.02	988.79	3.95	0.86	192.27	2.70	0.87	198.23
180	2.50	0.00	1000.0	5.30	0.82	232.52	3.00	0.87	220.40
200	2.65	0.04	959.34	6.80	0.78	272.15	3.50	0.85	265.71
